# Assessing the hydrolysis of PDO threads combined with injectable hyaluronic acid solutions for facial rejuvenation

**DOI:** 10.1590/0103-644020246220

**Published:** 2025-11-21

**Authors:** Flávia Pires Bretas Delaroli, Ana Paula Christakis Costa, Juliano Bacom Modesto Setti, Patrícia Moreira de Freitas, Victor Elias Arana-Chavez, Maristela Maia Lobo

**Affiliations:** 1 Postgraduate Program in Odontology. São Leopoldo Mandic College, Campinas, São Paulo, Brazil.; 2 Federal University of Technology - Paraná (UTFPR), Graduate School of Electrical Engineering and Applied Computer Sciences (CPGEI), Curitiba, Paraná, Brazil; 3 Department of Restorative Dentistry. Special Laboratory of Lasers in Dentistry. School of Dentistry - University of São Paulo. São Paulo, SP, Brazil; 4 Department of Biomaterials and Oral Biology. Postgraduate Program in Dentistry/Oral Biology. School of Dentistry - University of São Paulo, São Paulo, SP, Brazil.; 5 Associate Professor. Department of Postgraduate Program in Odontology, concentrated area of Orofacial Harmonization. São Leopoldo Mandic College, Campinas, São Paulo, Brazil

**Keywords:** Hyaluronic Acid, Polydioxanone, Cosmetic Techniques, Rejuvenation

## Abstract

Resumo

O presente estudo visa avaliar, in vitro, a hidrólise de fios de monofilamento PDO quando associados a 4 soluções diferentes: Grupo A (GA) com solução salina; Grupo B (GB) com ácido hialurônico não reticulado TKN HA - MW 2% (Toskani); Grupo C (GC) com NCTFTM. 135 HA (Filorga); e Grupo D (GD) com CN 160 (PHD from Brazil). Para este efeito, foram incubados e medidos 3 filamentos de fio PDO de 1,0 cm (monofilamento liso de 29G/38 mm com fio I, da MedBeauty) nos tempos: 0, 7, 14, 30, 45, 60 e 63 dias após o início do experimento. A hidrólise das amostras foi avaliada por espectrometria de absorção, microscopia óptica e microscopia eletrônica de varredura. Os dados foram analisados pelos testes estatísticos Kruskal-Wallis, Friedman e t-Student-Newman-Keuls pelos programas SPSS 23 (SPSS Inc., Chicago, IL, EUA) e BioEstat 5.0 (Fundação Manirauá, Belém, PA, Brasil), fixando o nível de significância em 5%. Os resultados da espectrometria mostraram que, até 14 dias, as diferentes soluções tiveram efeitos distintos na hidrólise de fios de PDO (p=0,023). Após 45 dias, as hidrólises da amostra não apresentaram diferenças significativas entre os quatro grupos (p=0.065), indicando que todas as soluções testadas tiveram um efeito semelhante nos fios de PDO. E depois de 63 dias, apenas grupos GA e GD fios permaneceram não-hidrolisados, enquanto os demais foram completamente degradados. Em conclusão, a associação dos fios de PDO com substâncias injetáveis para intradermoterapia à base de ácido hialurônico não reticulado (AH) influência na hidrólise dos fios ao longo do tempo.

## Abstract (en)

The present study aimed to evaluate, in vitro, the hydrolysis of PDO monofilament threads when incubated in 4 different solutions: Group A (GA) with saline solution; Group B (GB) with non-reticulated hyaluronic acid TKN HA - MW 2% (Toskani); Group C (GC) with NCTF^TM.^ 135 HA (Filorga) and Group D (GD) with NC 160 (PHD from Brazil). For this purpose, three filaments of 1.0 cm of PDO thread (I-thread monofilament smooth 29G/38 mm, from MedBeauty) were incubated and measured at times: 0, 7, 14, 30, 45, 60, and 63 days after the start of the experiment. The sample hydrolysis was assessed by absorbance spectrometry, optical microscopy, and scanning electron microscopy. The data were analyzed using the Kruskal-Wallis, Friedman, and Stundet-Newman-Keuls t-test using SPSS 23 (SPSS Inc., Chicago, IL, USA) and BioEstat 5.0 (Manirauá Foundation, Belém, PA, Brazil) software programs, with a significance level of 5%. The spectrometry results showed that up to 14 days, the different solutions had distinct effects on the hydrolysis of PDO threads (p=0.023). After 45 days, the sample hydrolyses did not present a significant difference between the four groups, indicating that all tested solutions had a similar effect on the PDO threads (p=0.065). After 63 days, only groups GA (p=0.029) and GD (p=0.042) threads remained non-hydrolyzed, while others were completely degraded. In conclusion, the PDO association with intradermic injectables based on non-cross-linked hyaluronic acid (HA) affects the hydrolysis of the threads over time.

**Figure:**
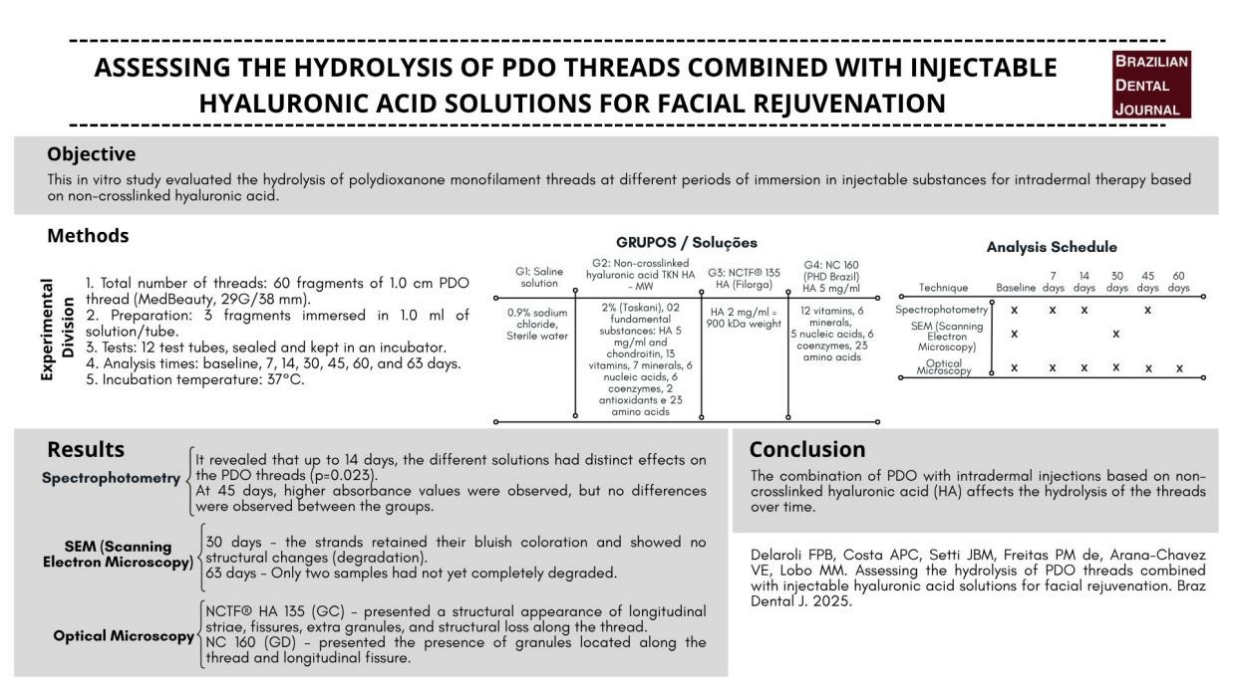

## Introduction

Skin aging is a multifactorial process characterized by wrinkles, loss of elasticity and tone, and dehydration ^1^. It considerably impacts people's appearance, especially at older ages ^1^. Exposure to ultraviolet radiation ^1^, accumulation of free radicals ^2^, and decreased biosynthetic activity of fibroblasts are the main factors in skin aging ^3^. In this context, the search for effective treatments to mitigate or reverse these signs has driven the development of various facial rejuvenation techniques focusing on mesotherapy and the application of monofilament polydioxanone (PDO) threads. It is known that the insertion of PDO threads into the dermis or subcutaneous tissue creates localized trauma that promotes neocollagenesis ^1^ ^,^ ^3^. Complementarily, hyaluronic acid plays a facial-filling role due to its high-water retention capacity, accompanied by minimal immunogenicity ^4^ ^,^ ^5^. Indeed, some studies show that mesotherapy can improve skin elasticity, complexion radiance, dermal thickness and density, as well as reduce wrinkle depth and pore size ^1^ ^,^ ^3^ ^,^ ^6^ ^,^ ^7^. Therefore, experts in orofacial dentistry have been using PDO thread application in combination with intradermal injection of actives such as hyaluronic acid ^1^ ^,^ ^6^ ^,^ ^8^ ^,^ ^9^.

PDO threads stand out as absorbable biomaterials ^10^, used in sutures and aesthetic procedures like facial rejuvenation. Studies show that these threads stimulate collagen synthesis and increase transforming growth factor (TGF-β1) ^8^ ^,^ ^11^. The properties of these threads include biocompatibility, gradual absorption, safety, durability, minimal inflammatory reaction, and proven efficacy in treating facial sagging and wrinkles ^8^ ^,^ ^10^ ^,^ ^12^. However, despite these benefits, it is important to note the lack of long-term studies on the safety and efficacy of these threads ^13^. Additionally, the thread fixation technique needs improvement to avoid displacement or granuloma formation ^11^.

Sabino and colleagues (2000) studied the hydrolytic degradation of absorbable polydioxanone through morphological changes and elasticity modulus. These experiments observed molecular mass changes and diffusion of lighter segments in the reaction medium ^14^. Another study conducted on absorbable monofilament threads (MonoFlex) showed hydrolytic degradation and a halving of their original strength after four weeks of incubation in PBS at 37°C. In rats, the suture material was completely absorbed after 180 days of implantation ^15^. De La Puerta and collabs. Conducted a comparative study of the mechanical properties and absorption of absorbable synthetic suture threads incubated in bovine serum. The results showed that degradation mainly occurs by hydrolysis through ester bond rupture ^16^. More recently, Bernardini (2019) compared the efficacy of lifting threads with traditional surgery for correcting facial wrinkles. The authors highlighted positive aspects of PDO thread use, such as patient satisfaction, quick recovery, and fewer complications associated with lifting, questioning the preference for traditional surgery over the efficacy and lower cost of thread procedures ^17^. However, Suárez-Vega et al. reported a case of a female patient with complications resulting from superficial implantation of barbed PDO threads in the midface region. Clinical manifestations included edema, bruising, superficial thread palpation, and skin puckering. The proposed treatment involved PDO thread degradation by injecting HA into the implant area, leading to reduced edema, puckering, and skin irregularities due to thread tension, as well as the attenuation of secondary wrinkles ^18^.

Siqueira & Canevassi (2022) also demonstrated the efficacy of combining smooth PDO thread application with permeation with NCTF 135 HA to stimulate facial collagen production, contributing to preventing biological aging effects. This drew attention to HA's role in inducing hydrolytic thread degradation, leading professionals to adopt this approach to intensify lifting effects. However, this procedure remains controversial as PDO threads are hydrophilic, and the presence of HA would accelerate the hydrolysis rate ^19^. Ultramicroscopy results revealed significant changes in fiber structure with increased spacing between layers and dilution of violet pigmentation after 24 hours of incubation, with these effects aggravated up to 72 hours, showing that non-crosslinked HA acts as a hydrolytic thread degradation catalyst ^20^. El-Domyati and colleagues (2012) conducted a clinical study of mesotherapy for periorbital wrinkles, not observing significant differences in clinical evaluations before and after treatment ^21^.

In general, it is evident that this topic has been extensively studied; however, the results of HA and PDO thread association exhibit significant variability. This variability can be attributed to the heterogeneity of materials, as well as the lack of standardization in experimental setups. In addition, there is little experimental evidence proving the long-term aesthetic benefits of this association. This is primarily due to the hydrophilic nature of HA, which can attract more water to the site of its application and interact with the ester bonds in the PDO polymer chain, resulting in the breaking of the material. In this context, the present work proposes to investigate the hydrolysis of monofilament PDO threads in the presence of common injectable intradermal substances based on non-crosslinked hyaluronic acid.

## Materials and methods

This in vitro study selected eighty-four smooth monofilament of polydioxanone PDO I-thread 29G/38 mm threads (MedBeauty) of 1 cm (p=0.05, CI 90%) and divided them into four groups (N = 3): group A (GA), incubation in saline solution; group B (GB), incubation in non-crosslinked hyaluronic acid TKN HA - MW 2% (Toskani); group C (GC), incubation in NCTF 135 HA (Filorga); and finally, group D (GD), incubation in NC-160 (PHD do Brasil). The composition of the items used in the study is shown in Table 1.

The experimental design of this in vitro study was structured to ensure statistical precision and operational feasibility when analyzing the degradation of polydioxanone (PDO) monofilament exposed to different bioactive solutions. The sample size was calculated using repeated measures analysis of variance (Repeated Measures ANOVA) with a significance level (α) of 5% (0.05) and statistical power (1-β) of 80%. These parameters are widely accepted in scientific literature as appropriate for detecting differences with statistical and practical relevance (Cohen, 1988). While the total number of filaments used (n = 84) may seem small compared to clinical trials, this is justified by a repeated-measures in vitro experimental model, in which each filament is evaluated multiple times throughout the study.

Three filaments were conditioned per group, and for each of the seven incubation times (0, 7, 14, 30, 45, 60, and 63 days at 37°C oven), the degradation of the PDO strands was monitored. These time points were selected to monitor the progressive degradation of the PDO strands over time to identify the critical points at which significant changes occur, such as collagen stimulation and enzymatic degradation of the strands. At each time point, hydrolysis of the threads was monitored by absorption spectrometry analysis at 400 nm (Epoch - Biotek) to evaluate the degradation of the threads by absorbance, optical microscopy from 4X to 100X magnifications (Balzers SDC-050) to observe the structural changes of PDO over time and blue color, and scanning electron microscopy (SEM, Leo 430), operating at 15 kV to analyze the structural change during hydrolysis.

At each of the incubation times, 0, 7, 14, 30, 45, 60, and 63 days, samples were measured and reincubated in the same solution. Optical microscopy analyses were conducted at 0, 7, 14, 30, 45, 60, and 63 days. Scanning electron microscopy (SEM) measurements were taken only on days 0 and 30. The purpose of these analyses was to verify the structural characteristics of PDO filaments in the different solutions. Spectrometry was performed on days 0, 7, 14, and 45. At these times, light absorption is proportional to the amount of substance absorbed.

Median differences were compared using the Kruskal-Wallis test, time effects were compared using the Friedman test, and multiple comparisons were performed using the Student-Newman-Keuls test. For this, the SPSS 23 (SPSS INC., Chicago, IL, USA) and BioEstat 5.0 (Fundação Mamirauá, Belém, PA, Brazil) programs were used, considering a significance level of p-value ≤ 0.05.

**Table 1:** Chemical composition of the intradermal substances of the HA.

Component	Saline solution	TKN HS - MW 2% (Toskani)	NCTF 135 HA (Filorga)	NC-160 (PHD Brasil)
General composition	Sodium chloride (NaCl) 0,9% (9 g/L Water q.s. to 1L pH 5.5 -7.0 Osmolarity ~308 mOsm/L			
Hyaluronic acid		High molecular weight (2%)	Non-cross-linked (5 mg/mL)	Provides hydration and volume
Vitamins			12 vitamins (A, B1, B2, B3, B5, B6, B8, B9, B12, C, E, K)	13 vitamins (A, B1, B2, B3, B5, B6, B8, B9, B12, C, D, E, K)
Amino Acids			24 amino acids (support collagen and elastin synthesis)	23 amino acids (essential for protein synthesis)
Coenzymes			6 coenzymes (catalyze biochemical reactions)	6 coenzymes (facilitate metabolic reactions)
Nucleic Bases/Acids			5 nucleic bases (support cell communication)	6 nucleic bases (aid in cell repair and replication)
Minerals			4 minerals (calcium, magnesium, sodium, potassium)	7 minerals (Zinc, iron, calcium, magnesium, sodium, potassium, phosphorus)
Antioxidants			2 Antioxidants (Glutathione, alpha-Lipoic acid)	2 Antioxidants (protect against free radicals)

## Results

**Figure 1:**
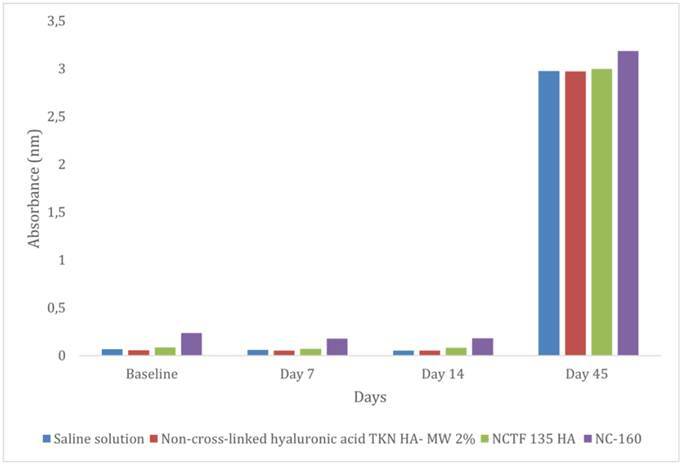
Bar plot of group median absorbance for each time point.

The absorption spectrometry results indicated that the hydrolysis of the threads within groups GA (saline solution) (0.052 nm; p = 0.029), GB (non-cross-linked hyaluronic acid TKN HA - MW 2%) (0.052; p = 0.042), and GC (NCTF 135 HA) (0.084; p = 0.049) showed statistically significant increases in absorbance over time up to day 14, although the magnitude of change was relatively modest. In contrast, the GD (NC-160) showed a more pronounced and statistically significant increase in absorbance (0.181; p = 0.042), suggesting an accelerated degradation process compared to the other three groups. By day 45, no statistically significant differences in absorbance were observed among the four groups, indicating a convergence in the hydrolysis behavior. However, temporal analysis revealed that, after day 45, all four solutions exhibited increased absorbance values compared to those observed on days 0, 7, and 14, indicating a progressive degradation. These results are detailed in Table 2 and illustrated in Figure 1.

**Table 2:** Absorbance median and SDs of the three measured samples for each solution and incubation time.

Intradermal therapy solution	day 0	day 7	Day 14	Day 45
	0.068	0.059	0.052	2.977
GA (Saline Solution)	(0.007) Aa	(0.003) Aa	(0.005) Aa	(0.038) Ab
	0.056	0.053	0.052	2.974
GB (TKN HA- MW 2%)	(0.002) Aa	(0.002) Aa	(0.003) Aa	(0.022) Ab
	0.085	0.073	0.084	2.997
GC (NCTF 135 HA)	(0.004) Aa	(0.006) Aa	(0.016) Aa	(0.029) Ab
	0.236	0.176	0.181	3.185
GD (NC-160)	(0.006) Ba	(0.010) Ba	(0.028) Ba	(0.050) Ab

Comparisons between groups: day 0, p = 0.016; day 7, p = 0.016; day 14, p = 0.023; and day 45, p = 0.065. Comparisons between time points: GA, p = 0.029; GB, p = 0.042; GC, p = 0.042; and GD, p = 0.049. Groups with medians followed by different uppercase letters differ significantly (comparisons between time points). The Kruskal-Wallis statistical test was used to ascertain the effect of the intradermal therapy solutions; the Friedman test investigated the effect of time, and the Student-Newman-Keuls test analyzed multiple comparisons. Means followed by lowercase letters indicate significant difference between times (comparisons between groups).

**Figure 2:**
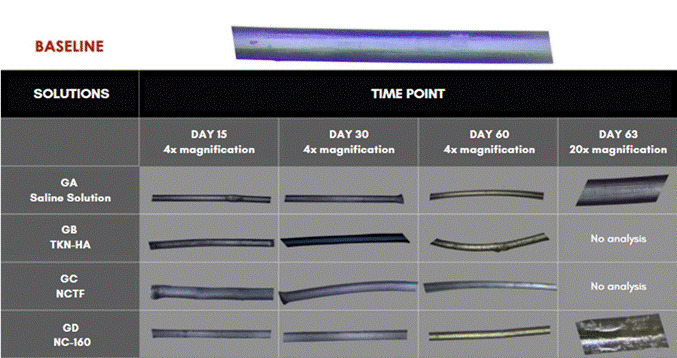
Microscopic images of PDO filaments incubated in different solutions at each time point, 4X and 20X magnification.

Optical microscopic analysis at 4X magnification showed that the structure of the PDO threads remained bluish until day 30. From that day on, it began to exhibit visible structural changes of filament degradation. By day 63, only the GA and GD samples were clamped. The others had degraded (Figure 2).

**Figure 3:**
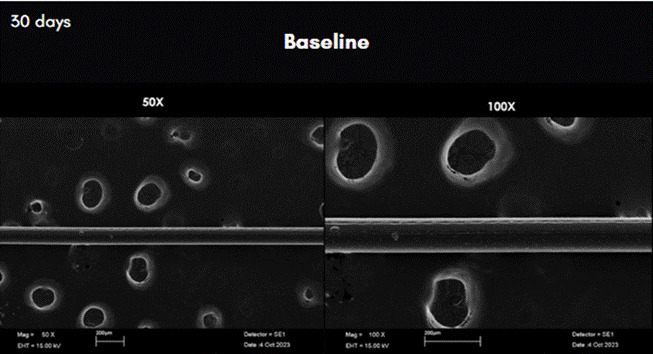
Scanning Electron Microscopy images of the row PDO threads incubated in saline solution after 30 days. Magnification: 50X on the left; and 100X on the right.

**Figure 4:**
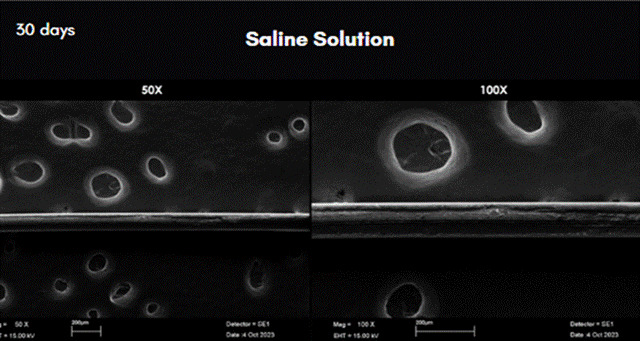
Scanning Electron Microscopy images of the row PDO threads incubated in saline solution after 30 days. Magnification: 50X on the left; and 100X on the right.

**Figure 5:**
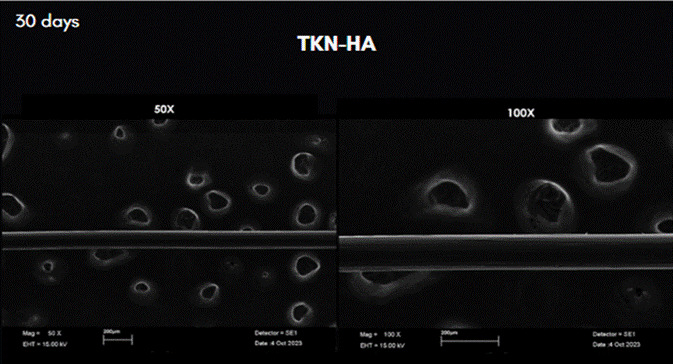
Scan Electron Microscopy images of the row PDO threads incubated in TKN-HA after 30 days. Magnification: 50X, on the left; and 100X on the right.

**Figure 6:**
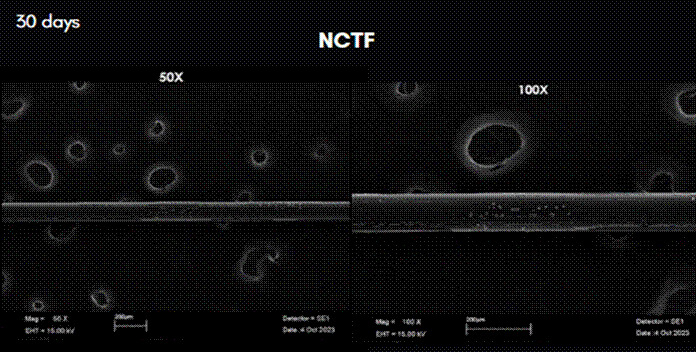
Scan Electron Microscopy images of the row PDO threads incubated in TKN-HA after 30 days. Magnification: 50X on the left; and 100X on the right.

**Figure 7:**
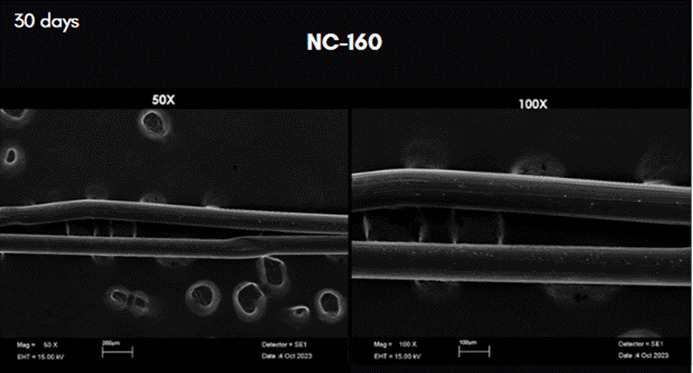
Scan Electron Microscopy images of the row PDO threads incubated in TKN-HA after 30 days. Magnification: 50X on the left; and 100X on the right.

The results of the analysis of scanning electron microscopy images can be observed in Figures 3 to 7. In these figures, the images were obtained from samples incubated after 30 days and are presented at 50X and 100X magnification. The comparative analysis between the threads of the GA group at day 0, incubated in saline solution, and the GC group at day 30, incubated in NCTF^TM^ HA 135, showed a noticeable alteration (Figures 4 , and 6). It can be observed that after 30 days, the threads exhibit a structural aspect of longitudinal streaks, fissures, extra granules, and structural loss along the thread.

At 30 days the SEM analysis on samples from GD (NC-160) exhibited granules and longitudinal fissures. Absorbance analysis at 14 and 45 days showed results similar to those of the other groups (Figure 7) 5.

## Discussion

The results obtained demonstrate that HA solutions influence the structural integrity of PDO threads, particularly over extended periods, as in this study. NC-160 was the most conservative solution in terms of thread degradation, while NCTF 135 HA induced significant changes, the desirability of which may vary depending on the therapeutic objective. Microscopic analysis complements the absorbance data by providing visual evidence of structural degradation and corroborating the statistical findings. This is supported by the findings of Ping Ooi and Cameron (2002) ^22^ and Suárez-Vega et al. (2019b) ^20^, who, using similar experimental setups, observed the first signs of alteration approximately 15 days after filaments incubation in HA, as found in this study. However, by 45 days, as in this study, the filaments exhibited degradation, aligning with the observations of Suárez-Vega et al. (2019a) ^18^, where the filaments experienced a loss in tensile strength. The study did not analyze the mechanical strength of the filaments when immersed in HA; therefore, it cannot be proven that the PDO threads investigated suffer a loss of tensile strength.

The absorbance analysis carried out led to the hypothesis that non-cross-linked HA is a catalyst for the hydrolytic degradation of polydioxanone since it is highly hydrophilic. That is, a bond is established between HA and the polymeric chains that compose the thread. Therefore, Ping Ooi and Cameron (2002) ^22^ and Suárez-Vega et al. (2019b) ^20^ in experiments like this one indicate that the first change occurs approximately after 15 days of thread immersion in HA, corroborating the results found in this study and disagreeing with those of Suárez-Vega et al. (2019a) ^18^.

The results of optical microscopy analysis showed that the threads kept their blue color unchanged without appreciable structural alterations resulting from degradation. The structural integrity of the threads, in turn, was maintained for 45 days of incubation in all groups, as reported in the experiments by Suárez-Vega (2019b) ^20^. However, an accumulation of HA was observed around the threads of the GB group, incubated in TKN HA, at times day 30 and day 60, and in the threads of groups GC and GD respectively incubated in NCTF HA 135 and NC-160, at day 60, all of them compared to their corresponding GA groups, in which the threads were incubated in saline solution. Also in this experiment, no swelling was observed in the filament structure of the threads, as reported in the experiments of Suárez-Vega et al. (2019b) ^20^. At day 63 of the experiment, only NC-160 and saline solution presented residues of PDO threads that could be pinched within the test tubes. In the other groups, the threads had already been completely degraded. Thus, different injectable solutions, such as saline solution and non-cross-linked HA, may affect the speed and extent of thread hydrolysis at different times.

The results of this in vitro investigation suggest that non-cross-linked HA-based solutions may influence thread hydrolysis. However, the results obtained from the analysis of PDO threads incubated in saline solutions indicate that the threads were equally degraded over time, presenting absorbance values very similar to those obtained from incubation in HA-based solutions at day 45. Saline solution, being a neutral solution (pH 7.0), acted as an inert medium, providing an environment in which the natural process of PDO hydrolysis could occur without obstacles. The role of saline solution in this context was to maintain an adequate environment to prevent PDO degradation, without chemical interference, providing comparative data to enable further analyses under different types of acids. An acidic environment could trigger an additional degradation autocatalytic effect ^14^. Sabino et al. conducted experiments in a phosphate buffer solution at pH 7.4, which is close to the pH of saline solution. The PDO thread degraded over 180 days of incubation. In the present study, the PDO threads immersed in HA- based solutions with pH levels similar to those of a phosphate buffer solution remained remained in the degradation process throughout the monitoring period (63 days), corroborating the conclusions of Sabino and collabs.

The association of PDO threads with non-cross-linked TKN HA, NCTF 135 HA, and NC-160 demonstrated the presence of hydrolysis over time. However, there was no significant difference between the groups regarding hydrolysis after 45 days. This reaction may result from the interaction of HA molecules with PDO fibers through physical entanglement and hydrogen bonds. This interaction may delay the hydrolysis process to some extent, as it can create a protective barrier around the PDO thread, forming a gelatinous and hydrated layer for subsequent degradation ^19^. As in NCTF, these reactions can be seen in the NC-160 group. Regarding TKN HA, the threads showed significant hydrolysis with increasing absorbance values over time, without significant differences in the first 15 days of the experiment.

## Conclusion

In conclusion, the PDO association with intradermic injectables based on non-cross-linked hyaluronic acid affects the hydrolysis of the threads over time. As this is in vitro, the results may not reflect physiological conditions and may be influenced by thread degradation in an intradermal environment. Additionally, evaluating only three HAs under controlled conditions may limit the results and prevent extrapolation to other products. The 63-day monitoring may have limited the study, as it was considered short in comparison to other studies that evaluated for 180 days. It is worth noting that the threads have already degraded by 63 days.

The results should be considered to improve rejuvenating aesthetic protocols based on HA. Moreover, more in vivo studies of PDO thread degradation should be encouraged to improve such a combination of treatments with other analysis techniques.
